# Whole-genome cancer analysis as an approach to deeper understanding of tumour biology

**DOI:** 10.1038/sj.bjc.6605497

**Published:** 2009-12-22

**Authors:** R L Strausberg, A J G Simpson

**Affiliations:** 1 J Craig Venter Institute, 9704 Medical Center Drive, Rockville, MD 20850, USA; 2 Ludwig Institute for Cancer Research, 605 Third Avenue, New York, NY 10158, USA

**Keywords:** genome, transcriptome, exome, chromosome, sequencing

## Abstract

Recent advances in DNA sequencing technology are providing unprecedented opportunities for comprehensive analysis of cancer genomes, exomes, transcriptomes, as well as epigenomic components. The integration of these data sets with well-annotated phenotypic and clinical data will expedite improved interventions based on the individual genomics of the patient and the specific disease.

The family of diseases that we refer to as cancer represents a field of application for genomics of truly special importance and opportunity. It is perhaps the first area in which not only will genomics continue to make major contributions to the understanding of the disease through holistic discovery of causal genome-wide perturbations but will also be the first field in which whole-genome analysis is used in clinical applications such as precise diagnosis, prognosis and prediction of response to treatment. There are several reasons that underlie the excitement due to access to whole-genome DNA sequencing. First, the altered cancer genome is the direct cause of disease and precisely defines the tumour phenotype. Second, as we have access to both diseased tissue and normal samples from the same patient, and as most cancer genomic alterations represent somatic events, we can discern with confidence those changes specific to cancer. Third, in cancer, genomic alterations are progressive and in some cancer cases, changes related to disease stage, development of metastases and drug resistance are discernible. Thus, the increasing sophistication of systems biology now means that the complex interplay of events that activate and inactivate specific genes and pathways can be deduced directly from deep genomic and transcriptomic sequencing (Figure 1). In this overview, perspective is provided with respect to the approaches that have been developed over the past decade and emerging opportunities for discovery and eventual application.

Starting in the late 1990s, much of the gene- and transcriptome-based data were provided through large-scale community resource projects such as the NCI's Cancer Genome Anatomy Project, the Sanger Centre's Cancer Genome Project and the Human Cancer Genome Project of the Ludwig Institute for Cancer Research (Strausberg *et al*, 2003). These projects complemented the growing knowledge of specific gene alterations discovered by the cancer research community and provided a basis for more global views of tumourigenesis, on the basis of knowledge of gene families and signal transduction networks. More recently, technological changes in DNA sequencing, including the recently introduced ‘NextGen’ instruments and associated molecular technologies, have enabled both higher-throughput and more sensitive assays that provide important new opportunities for basic discovery and clinical application (Mardis, 2009). The new technologies also provide for more democratised large-scale DNA sequencing that will facilitate enhanced cancer genomics opportunities for the cancer research community within both tumour biology and clinical laboratories.

Recently, the first genomic sequence of a cancer (a case of acute myeloid leukaemia (AML)) was described by Ley *et al* (2008) in a landmark publication, an important advance in our knowledge of the complete molecular repertoire of cancers.

## Towards an era of whole-genome cancer sequencing

In the report by Ley *et al*, which describes the complete genome sequencing of AML, Illumina genome analyser (GA) technology was used to attain a nearly 33-fold coverage of the genome. In addition, a 14-fold genome coverage of a normal skin sample from the same patient was obtained. The cancer genome of this individual is cytogenetically normal and diploid, representing a far simpler case than the genomes of cytogenetically complex and much more common carcinomas. Nevertheless, the analysis provides an informative snapshot of what we can expect from whole-cancer genome shotgun sequencing. The central focus of this genome project is the detection of somatic mutations, the principal driving force in cancer.

Of the somatic mutations detected, more than 11 000 were located within annotated genes. However, most (more than 10 700) were in introns and another ∼200 were in non-translated regions. These were not analysed further for possible functional significance. Of the 181 potential somatic mutations encoding alterations in protein sequence, many were eventually eliminated from further consideration. For example, 152 were determined to be false positives, and others were in inherited SNPs or were not confirmed by PCR. Gene-based analysis of the remaining variants revealed a total of 10 that are predicted to result in an altered protein sequence. Of these, two are in genes that have already been shown to recurrently carry mutations in this tumour type, *FLT3* and *NPM1,* and eight are therefore newly described and of unknown function or relevance to the tumourigenic process. On the basis of studies in additional samples, however, there was no evidence to suggest that these additional eight genes are recurrently mutated in AML. Although recurrence would suggest that mutations do contribute to tumourigenesis, the absence of recurrence does not necessarily mean that they do not. Although informatics analysis reduced the number of variants of specific interest to a relative few, large numbers of additional potentially functional alterations that could potentially contribute to the cancer repertoire were also identified, but not deemed accessible for further functional consideration at this time (and the potential role of insertion/deletion changes was not considered).

In a subsequent study, Mardis *et al* (2009) performed Illumina whole-genome sequencing on a second AML, as well as on a skin sample derived from the same patient. Of the 52 somatic point mutations detected, four were recurrent in at least one other sample. Two of the four alterations were previously unknown in AML, both with interesting characteristics. In the first instance, mutations were discovered in the *IDH1* gene, at the same position as in glioblastoma, but with different preferences with respect to the coding change. These alterations were most prominent in AML with normal diploid karyotypes. The second alteration, found in an additional AML sample, is located in a non-genic evolutionarily conserved region, pointing to the importance of developing a strategy for informing analysis of non-protein encoding alterations with potential regulatory functions. Indeed, genome-wide association studies also point to inherited non-genic alterations in cancers (De Gobbi *et al*, 2006; Steidl *et al*, 2007; Easton and Eeles, 2008). Understanding the potential regulatory effects of these alterations that are currently not understood will be of key importance in understanding the molecular mechanisms of cancer. In that regard, progress of the ENCODE project (Birney *et al*, 2007) will be beneficial towards providing a platform for providing a context towards assessing the role of non-genic alterations in cancer development and progression.

A recent whole-genome analysis of a lobular breast cancer sample points to informative features of cancer progression discernible with NextGen sequencing (Shah *et al*, 2009). In this, an Illumina sequencing-based approach was used to sequence the genome of a primary tumour, as well as a metastasis collected from the same patient 9 years later. The results provided insight into the evolution of the cancer genome associated with disease progression. For example, of the 32 somatic alterations detected in metastasis, only 11 were detected in primary tumour. Moreover, the digital characteristics of NextGen sequencing revealed frequency differences among the 11 alterations detectable in primary tumour, with some being very prevalent in the tumour and others detectable in as few as 1% of tumour genomes, thereby reflecting differences in the appearance of changes and/or their prevalence with specific cell types. As the authors note, the prevalence of new mutations in metastases could reflect those associated naturally with tumour progression, as well as those induced by treatments such as radiation therapy.

A second very notable feature of the study by Shah *et al* is the integration of genome and transcriptome analysis. With the precise view of each facilitated by NextGen sequencing, new insights into the repertoire of tools accessible to potentially drive cancer progression are discernible. In this case, several hundred putative RNA-editing events were observed that would potentially result in non-synonymous protein changes not coded directly by the gene. Non-synonymous editing events were confirmed in the *COG3* and *SRP9* genes, and it was also noted that the *ADAR* gene, encoding a key RNA-editing enzyme, is highly expressed in this cancer. Together, these results highlight the major theme of the importance of data integration, and that the quantitative and digital aspects of NextGen sequencing can together be applied to an understanding of gene activation/inactivation. This combined approach thereby provides greater insight into the similarities and differences of related cancers, and how these features can guide targeted intervention not only based on specific genetic alterations but also on all alterations that can contribute to an alteration of targeted genes and pathways.

These first cancer genome sequences point to the challenge of functional studies of large numbers of variants of unknown function. In keeping with that notion, several studies have focused on sequencing the entire cancer exome (the complete set of exons) or selected sets of exons in gene families.

## The current focus on the cancer exome

Using the platform of the completed human genome enabled the cancer research community to pursue the targeted sequencing of gene families, starting initially with tyrosine kinases (Davies *et al*, 2002; Bardelli *et al*, 2003) and progressing recently to almost the entire human gene content in selected cancers, including breast and colorectal (Wood *et al*, 2007), pancreas (Jones *et al*, 2008) and glioblastoma (Parsons *et al*, 2008). These studies have provided examples of the enhanced value gained through comprehensive analysis compared with gene-by-gene approaches. The kinome was an attractive gene family for initial studies, given the successful introduction of new cancer therapies, such as Gleevec (Novartis Pharmaceuticals Corporation, East Hanover, NJ, USA; Druker, 2008), that target kinases activated in cancers. These studies were informative in multiple ways. First, the initial report of Bardelli *et al* (2003) pointed to the large number of tyrosine kinase genes mutated in individual colorectal cancers, suggesting that, although most of these genes carry somatic mutations in a limited number of tumour samples, together these genes are mutated in a high proportion of colorectal cancers. Moreover, from these studies, frequent mutations in particular genes were discovered, including *BRAF* in malignant melanoma (Davies *et al*, 2002) and *PIK3CA* in colorectal and other cancers (Samuels *et al*, 2004), thus identifying potentially important new targets for therapeutic intervention.

The more recent comprehensive breast and colorectal cancer studies, comprising the exons of the best-characterised human genes including those in RefSeq (http://www.ncbi.nlm.nih.gov/RefSeq/), pointed to the presence of a relatively few genes with somatic mutations in a high proportion in those cancers, and a much higher number of genes that are mutated relatively infrequently. This pattern of mutational frequencies means that the permutation of mutations in any individual tumour is essentially unique. However, much greater consistency and a clearer picture emerges if the mutational patterns are considered in the context of biological pathways, leading to the identification of both common and variable features of cancers (Leary *et al*, 2008). Very informative to this analysis will be the integration of various data sets towards the definition of the pathways and networks that drive cancers. Complementary to the study of genomic changes are the fluid alterations of the coding repertoire, such as those represented in gene expression levels and the specific transcript forms that are expressed. As was noted by Shah *et al* (2009), integrating these quantitative and qualitative changes in cancer cells and assessing the changes that actually have functional consequences become a key issue going forward.

Much of the exome data to date comes from Sanger sequencing that can best detect those mutations that are clonal within the tumour, for technical reasons. The use of NextGen technologies for deeper exome sequencing will now offer the potential to discern rare events within a tumour or a particular cell type, as the new technologies are based on amplification of individual molecules. A most interesting example of this approach was provided by a study on *EGFR*-based Gefitinib resistance, which demonstrated resistance based on mutations that were not clonal, but could be revealed by application of the 454 Sequencing System (454 Life Sciences, Branford, CT, USA; Thomas *et al*, 2005).

## The importance of integrated data sets

The whole-cancer exome projects point to the importance of measuring various forms of perturbational alterations of genes, including both exonic point mutations and insertion/deletion events, in assessing the relative potential contribution of any gene to cancer. In the study by Parsons *et al* (2008), the two most commonly altered genes in glioblastoma are predominantly altered through quite different mechanisms (homozygous deletions for *CDKN2A* and point mutations for *TP53*). Moreover, detailed views of altered glioblastoma pathways, including *TP53*, *PI3K* and *RB1*, revealed the presence of point mutations, amplifications and deletions in each instance. This study also pointed to patterns of coincidental and exclusive mutations. For example, the newly identified *IDH1* mutation was often coincidental with *TP53* mutations (83% of cases compared with 27% in patients with wild-type *IDH1*) and negatively correlated with the presence of *PTEN*, *RB1*, *EGFR* or *NF1* mutations (0% of cases compared with 60% in patients with wild-type *IDH1*), suggesting fundamentally different cancers at the molecular level. As these patterns are more extensively studied in the context of tumour progression and response to therapy, clinical sequencing will become increasingly useful as a tool for the attending oncologist.

The comprehensive study of glioblastoma also points to the importance of using different strategies in delineating phenotypic correlations. This cancer was also selected in The Cancer Genome Atlas pilot study in which fewer genes were initially sequenced, but in a deeper sample set (Cancer Genome Atlas Research Network, 2008). The study further showed the importance of integration of different analytical approaches, in this instance, gene expression and epigenomic data. This study identified an additional frequently mutated gene, *PIK3R1*, as well as a strong association of the methylation status of the *MGMT* promoter and frequency of G–C to A–T transitions within CpG sites compared with non-CpG dinucleotides. The recognition that the cancer genome is more than that of the As, Cs, Ts, and Gs through the incorporation of methylation and additional epigenomic information will be an increasingly important and commonplace approach while going forward with NextGen sequencing. Technologies such as Chip-Seq open new avenues for research, such as in the study of enhancer-associated regulatory protein binding sites (Visel *et al*, 2009), as well as to assess the specific relationships of specific histone modifications as they relate to features such as transcription factor binding (Robertson *et al*, 2008; Gargiulo and Minucci, 2009; Gargiulo *et al*, 2009; Neff and Armstrong, 2009).

## Cancer genome rearrangements – an important component of the cancer repertoire

Rearrangements of the cancer genome, including amplifications/deletions and chromosomal translocations, represent a biologically and clinically important, but poorly characterised, class of somatic variation in cancer. This is especially true in common carcinomas in which cytogenetic patterns are often very complex. However, cancer researchers clearly recognise the importance of these events, especially as they led to the development of two early successfully targeted cancer therapies, Herceptin (Genentech, Inc, South San Francisco, CA, USA) (based initially on amplification of *ERBB2* in breast cancer) (Park *et al*, 2008) and Gleevec (Druker, 2008), based on *BCR–ABL* translocation in chronic myelogenous leukaemia.

Advances in sequencing technology currently have a major impact in illuminating the molecular identity of these events (Chiang *et al*, 2009). The very cost-effective deep genome coverage of NextGen sequencing facilitates a quantitative detection of regions that are over- or underrepresented, compared with a reference genome, and that constitute potential regional amplifications or deletions. In addition, the recent incorporation of paired-end sequencing in NextGen platforms allows the detection of insertions/deletions and also translocations based on differential mapping of the paired-end reads in comparison with the reference genome.

Initial applications of these approaches have been quite successful (Bignell *et al*, 2007; Campbell *et al*, 2008). For example, Campbell *et al* (2008) used Illumina GA technology to attain the precise sequence of several hundred variants, comprising germline and somatic intra- and inter-chromosomal rearrangements in two lung cancer cell lines. The quantity and precision of the data sets point to the molecular derivation of such rearrangements including the role of retrotransposons. They also identified translocation events that result in the generation of novel fusion genes and transcripts. A deep study of genomes at this level will now begin to delineate those long-range alterations that are recurrent and associated with specific clinical features.

## The cancer transcriptome

Transcriptome analysis has been a major driver in the comprehensive molecular characterisation of cancer through expressed sequence tag sequencing, as well as tag-based approaches including SAGE. However, even the most imaginative approaches were of somewhat limited use because of the depth of sequencing and the information content of sequence tags. NextGen sequencing incorporating much deeper sequencing now provides glimpses of the rich information content that can be gleaned from the transcriptome, including not only alternative splice forms and non-protein encoding transcripts but also genomic alterations present in transcripts, such as somatic point mutations and gene alterations in fusions and truncations (Morin *et al*, 2008; Sugarbaker *et al*, 2008; Maher *et al*, 2009; Zhao *et al*, 2009).

For example, Sugarbaker *et al* (2008) described a deep analysis of the malignant pleural mesothelioma transcriptome on the basis of over 260 Mb of cDNA sequence generated by 454 Life Sciences sequencing. The study revealed alternative splice forms, new point mutations and small deletions resulting in non-synonymous changes, as well as variants derived from RNA editing. Although each mesothelioma had a different mutational profile on the basis of transcripts, several of the newly identified mutations were observed in multiple samples.

Two recent studies revealed additional information to be gleaned from deep-transcriptome sequencing. In these studies (Maher *et al*, 2009; Zhao *et al*, 2009), the transcriptomes of prostate and breast cancers were analysed for the presence of gene fusion events that result from chromosomal translocations. In the study by Zhao *et al* (2009), the pseudotetraploid breast cancer cell line HCC1954 was studied in comparison with a non-cancer-derived cell line from the same patient. Through mapping of ∼500 000 454-derived reads of RefSeq genes, eight novel gene fusion events were detected that reveal complex molecular events that result in fusion and truncated proteins, several in genes previously implicated in cancer. Subsequent verification by PCR and FISH of genomic DNA confirmed the genomic nature of these events.

The approach of Maher *et al* (2009) further demonstrated the use of deep-transcriptome sequencing (in this case, a combination of 454 Life Sciences long reads and Illumina GA short reads were used) to identify potential fusions, with initial proof of principle being to rediscover the *BCR–ABL* fusion in CML and the recurrent *TMPRSS2-ERG* gene fusion in prostate cancer. Several additional prostate cancer gene fusions were identified, although most are apparently non-recurrent. Interestingly, the only identified recurrent transcript that encoded a fusion protein *SLC45A3–ELK4* was not the result of genomic alteration, but apparently the result of a read-through transcription. This result further points to the still new and surprising means in which transcripts are generated to meet cellular needs, some being hard-wired in the genome, and others based on mechanisms not fully understood but adding to the repertoire of genome dynamics.

Finally, the importance of the non-coding transcriptome in cancer has been a focal point of attention over the past several years. Even now, new non-coding transcripts with potential regulatory functions are being discovered through the NextGen technologies. A recent example is the study by He *et al* (2008), in which the human anti-sense transcriptome was characterised by a tag-based approach on the Illumina GA instrument (Illumina, Inc, San Diego, CA, USA). Evidence for anti-sense transcripts was observed for over 6000 human genes pointing towards potential roles in gene expression, again pointing to the value of deep sequencing approaches for establishing fundamental components of the transcriptional apparatus.

## It is all in the biology

Cancer research has been transformed over the past decade by comprehensive molecular analysis, resulting in a much greater understanding of the molecular diversity of cancers, as well as common features in seemingly very different cancers. Soon, whole-cancer genome sequencing will become routine, bringing increased opportunities for invigorating basic discovery and also to make an impact on patient care. However, substantial challenges remain before the full potential on the latter can be realised.

As is apparent from recent studies, as well as the sum of research over the last decade, the numbers of alterations in the cancer genome, especially in solid cancers, are vast. Moreover, our knowledge of the repertoire of components that help to orchestrate cancer transitions has increased in surprising ways, especially in the context of the diversity and functional roles of non-coding RNAs.

Evident from current results and discussions is the challenge of understanding the diversity of genomic variants being discovered and their potential functional roles in cancer (Maher, 2009). Most analysis still focuses on the exons of cancer genes, and therefore both exon-based sequencing and transcriptome analysis will attain increased focus going forward and is likely to represent the dominant near-term strategy.

Cancer represents a special opportunity not only to study disease genesis and progression but also to study mechanisms of cellular regulation, as, for most cancers, both diseased and normal tissues are accessible from the same patient. We can thus identify specific genomic alterations in cancer compared with normal cells. Therefore, as much of cancer change is somatic, the variants that are causative to cancer will be discernible, although within an extensive background of variation that is not causative but is present as a result of the age-related disruption of the genome.

Within this context, we are fortunate to benefit from molecular organising principles of cancer that emerged even before the era of genome-wide technologies. Therefore, we have a framework of the functions that cancer cells must, in common, achieve (Hanahan and Weinberg, 2000), insights into the pathways and networks involved as the disease progresses (Jones *et al*, 2008) and the diversity of cells that contribute to cancer development, including to the cancer microenvironment (Weigelt and Bissell, 2008) and immune system (Dunn *et al*, 2004). Therefore, although the number and types of variants are large, and often not understood, we do possess a strong context within which to consider newly discovered features of cancer, as we fill-in the overall picture of cancer. The results of exome analysis to date are somewhat reminiscent of genome-wide association studies of other complex diseases in which many genes have a role, and many variants are likely to be quite rare. However in cancer, rare somatic variants in diseased tissue can be considered within the framework of known pathways and networks.

There is strong anticipation that NextGen genomics will lead to NextGen cancer care. Cancer genome sequencing is rapidly becoming more cost-effective such that we can envision this becoming a standard approach in tumour analysis. Indeed, the cost of the second AML genome was ‘dramatically’ less than the first, even with greater genome coverage (Mardis *et al*, 2009). With ongoing technological advances in DNA sequencing that will dramatically increase the throughput and reduce the cost, coupled with enhanced funding for cancer genome sequencing (Kaiser, 2009), our knowledge of human cancer genomes will increase dramatically over the next several years. Translating that sequence information to biological knowledge of cancer represents the key opportunity and challenge while going forward. Very important for driving biological knowledge and assessing how effective current advances are towards patient benefit are the current efforts to integrate genomic analysis within the clinical setting. We can certainly expect that a much stronger platform for informing cancer intervention will emerge on the basis of these efforts.

## Figures and Tables

**Figure 1:**
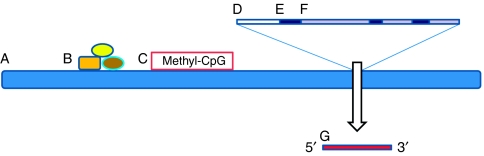
Current targets for genome-wide analysis by high-throughput sequencing. (A) Whole-genome through shotgun sequencing; (B) regulatory protein binding sites such as through Chip-seq; (C) epigenetic alterations including DNA methylation; (D) regulatory sites including promoters; (E) exons from selected genes or whole-exome sequencing; (F) intronic regions within genes; (G) the transcriptome including coding and non-protein-encoding transcripts (ncRNAs) and microRNAs.
